# Co-cultivation of *Lactobacillus zeae* and *Veillonella criceti*for the production of propionic acid

**DOI:** 10.1186/2191-0855-3-29

**Published:** 2013-05-24

**Authors:** David Dietz, Wael Sabra, An-Ping Zeng

**Affiliations:** 1 Institute of Bioprocess and Biosystems Engineering, Hamburg University of Technology, Denickestr.15, 21071 Hamburg, Germany; 2 Permanent address: Microbiology Department, Faculty of Science, Alexandria University, Alexandria, Egypt; 3 Present address: Fraunhofer Institute of Applied Polymer Research, , Geiselbergstr. 69, 14469 Potsdam, Germany

**Keywords:** Propionic Acid, Lactic Acid, *Lactobacillus zeae*, *Veillonella criceti*, Dialysis chamber reactor, Co-culture, Fermentation

## Abstract

In this work a defined co-culture of the lactic acid bacterium *Lactobacillus zeae* and the propionate producer *Veillonella criceti* has been studied in continuous stirred tank reactor (CSTR) and in a dialysis membrane reactor. It is the first time that this reactor type is used for a defined co-culture fermentation. This reactor allows high mixing rates and working with high cell densities, making it ideal for co-culture investigations. In CSTR experiments the co-culture showed over a broad concentration range an almost linear correlation in consumption and production rates to the supply with complex nutrients. In CSTR and dialysis cultures a strong growth stimulation of *L. zeae* by *V. criceti* was shown. In dialysis cultures very high propionate production rates (0.61 g L^-1^h^-1^) with final titers up to 28 g L^-1^ have been realized. This reactor allows an individual, intracellular investigation of the co-culture partners by omic-technologies to provide a better understanding of microbial communities.

## Introduction

Today most industrial processes are mono-culture processes due to a high degree of control, reproducibility and predictability. But mixed cultures arouse more and more interest. The high potential of mixed culture fermentations for industrial applications has been recently reviewed ( Bader et al. (2010), Sabra et al. (2010)). The authors stress the advantages of mixed cultures beside others in respect of wide substrate and product spectrum. These advantages may be applied e.g. in the fields of food and bioenergy.

Besides their technological relevance microbial communities have been investigated over the past decades due to their biological relevance in nature. Usually no mono-culture will occur in natural environments. In fact there exist complex microbial communities as biofilms that play an important role e.g. in human health ( Bryers (2008)).

One approach in understanding such complex communities is to investigate a defined part of this community, i.e. a defined co-culture of two organisms. In fact there exist a lot of studies with defined co-cultures including two microorganisms (e.g. Gerritse et al. (1990), Mikx and Vanderhoeven (1975), Tatton et al. (1989)). To have reproducible results for metabolic analysis experiments in a continuous stirred tank reactor (CSTR) are preferred. Usually substrate-limited conditions have to be investigated to prevent a wash-out of one organism, beside that a general problem of these studies is that no individualized study of both organisms is possible. To analyse the growth behaviour in defined co-cultures in a single reactor is possible with sophisticated molecular-biology methods (e.g. Schmidt et al. (2007)), but an individualization in different compartments would be preferable to investigate also intracellular processes.

To solve the latter problem, membrane-associated separation of the organisms in one study has been realized ( Egland et al. (2004)). In fact it was shown that bacteria do not only communicate by direct cell-cell-contact but also via low molecular metabolites that may pass through dialysis membranes ( Egland et al. (2004), Kolenbrander et al. (2010)). One approach to investigate a co-culture with separating the cells by a membrane is involving two reactors connected with each other by a membrane module. Manjarrez et al. (2000) used a hollow fiber module to investigate the amensalistic-type interaction between two *Saccharomyces* strains. They stress the superiority of this type of system over the EcoloGen system of New Brunswick Scientific (Edison, NJ, USA) described in Tannenbaum and Kornfeld (1974). The advantage is that in their hollow-fiber module system high mixing rates can be established that are important for fast interactions. But a problem for hollow fiber modules comes with their tendency to get blocked at higher cell densities.

The dialysis membrane reactor (Poertner and Maerkl (1998)) solves both problems: High mixing rates and high cell densities can be reached. In fact, up to now it was mostly used for high cell density fermentations (e.g. Markl et al. (1993)). The use and advantages of such a system for the investigation of a defined co-culture have been described ( Pestchanker and Ercoli (1997)), but to our best knowledge not realized up to now.

A mixed culture approach with industrial applicability is propionic acid production. *Lactobacillus zeae* and *Veillonella criceti* have been described as a defined co-culture for propionate production with a high potential in the industrial environment ( Mays and Fornili (1985), Sabra et al. (2012)).

In this work we use the dialysis membrane reactor for a defined co-culture for the first time with the example of the aforementioned co-culture. *L. zeae* will convert glucose to lactate which is the substrate of *V. criceti*, producing propionate and acetate. *V. criceti* is not able to use carbohydrates as a substrate, this leads to a commensalistic co-culture. We show the high potential for metabolic interaction studies with the membrane reactor and discuss about the possibility of the defined co-culture for propionic acid production in a CSTR process. So far these studies have mostly been performed with *Propionibacterium* species. It was a goal to find out cultivation conditions for this coculture to reach a high propionate concentration and to evaluate its feasibility for an industrial application.

## Materials and methods

### Strains and media

*Veillonella criceti* (DSM20734) and *Lactobacillus zeae* (DSM20178) were obtained from DSMZ (German Collection of Microorganisms and Cell Cultures, Braunschweig). The media for anaerobic precultivation contained KH_2_PO_4_ 15 g L^-1^, K_2_HPO_4_ 5 g L^-1^, Cystein.HCl × H_2_O 0,5 g L^-1^, peptone 2 g L^-1^, yeast extract 2 g L^-1^. For *V. criceti* pottasium lactate 10 g L^-1^ and resazurin 1 g L^-1^ were added. Anaerobiosis was achieved by nitrogen sparging and sealing with butyl-rubber stoppers of the serum bottles (50 mL medium in 100 mL bottles). After autoclaving, Na_2_S × 9 H_2_O 1 mM was added for *V. criceti*, for *L. zeae* glucose 10 g L^-1^ was added. Precultivation and fermentation were performed at 37°C, pH 6.0.

Fermentation medium was basically of the same composition, but had varying amounts of complex nutrients and carbon source as mentioned in the text. Yeast extract and peptone were added simultaneously in equal amounts to result in a certain concentration (g L^-1^).

### Fermentation

For CSTR fermentations, a 2l foil fermentor from Bioengineering (Wald, Switzerland) was used which is equipped with temperature and pH control. The fermentor was sparged before inoculation with nitrogen for at least 30 min. For titration, a 5 M NaOH solution was used.

**Figure 1:**
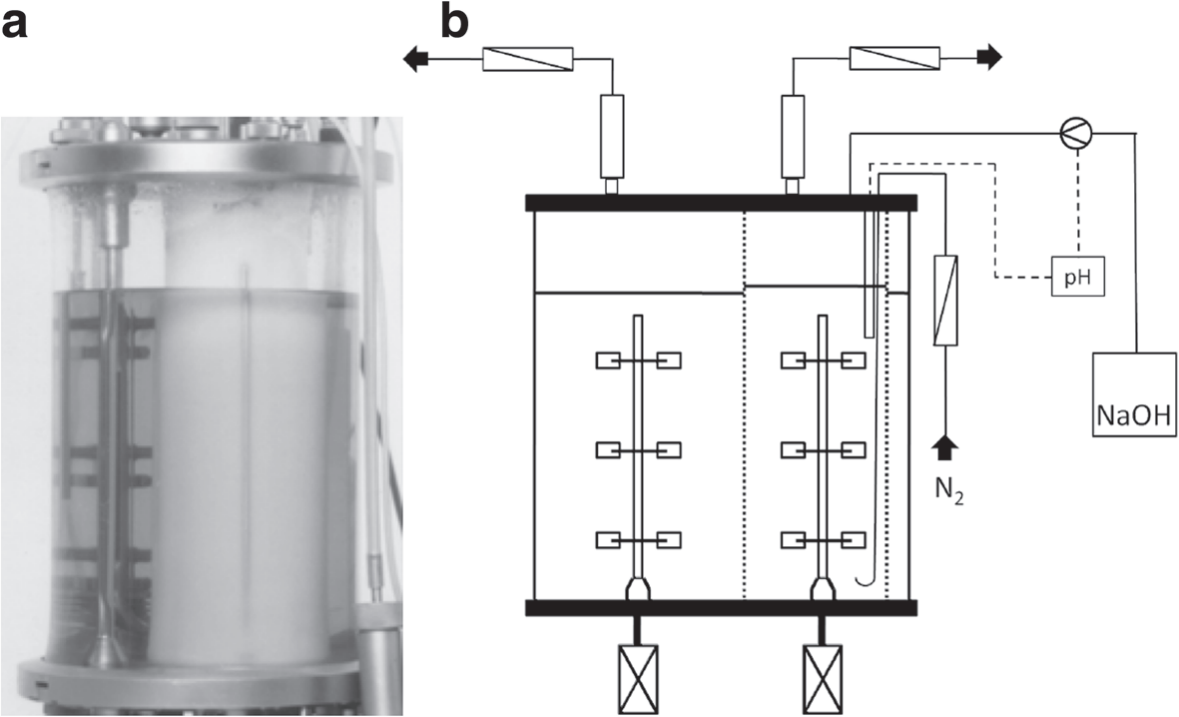
**(a) Picture of the running membrane dialysis reactor from**Poertner and Maerkl (1998**).** (**b**) Set-up of the dialysis reactor for the defined co-culture. The “inner chamber” (shows a higher liquid level) is separated from the “outer chamber” by a diffusion membrane. For more details see Poertner and Maerkl (1998**).**

For the co-culture experiments in the dialysis reactor a 7l foil fermentor from Bioengineering (Wald, Switzerland) was used (Figure 1). The working volume was 5 l (inner chamber 1.1 l, outer chamber 3,9l). The inner chamber has a Cuprophan-made membrane jacket (Table 1) with a 10 kDa cut-off, that allows diffusion of soluble medium components and metabolites over the membrane. For more details on this system see Poertner and Maerkl (1998).

**Table 1:** **Properties of the Cuprophan-membrane (Poertner and Maerkl (**1998**))**

	
Type of membrane	Non-porous diffusion membrane
Material	Regenerated cellulose
Thickness (*μ*m)	42
Area (cm^**2**^**)**	1570
Retention capacity (kDa)	10
Hydraulic permeability	0.004
(mL water min^**-1**^ **cm**^**-2**^ **bar**^**-1**^**)**	
Permeability coefficient for	0.094
glucose^*a*^ **(dm h**^**-1**^**)**	

^**a**^ Determined at T = 30 ***°***C, stirrer speed outer chamber 1500 rpm, inner chamber 1000 rpm.

### Analytics

Optical density of the cells was measured at 600 nm. Samples were filtered through 0.2 *μ*m filters. Glucose, lactate, propionate and acetate were measured by HPLC (Kontron, Germany) with an Aminex HPX-87H column (300 × 7.8 mm) at 60°C and with UV and RI detectors. H2SO4 (5 mM) was used as the mobile phase.

## Results

### CSTR experiments

First coculture experiments in a common stirred tank reactor were performed. Experiments started with a low concentration of complex nutrients, carbon source and a low dilution rate. Successively the glucose concentration was increased to reach higher product concentrations. Since nutrient limitations could be expected for these organisms, complex nutrients should be adapted to improve growth of one or both bacteria. Finally, by raising the dilution rate, the productivity of the process should be enhanced.

Cultivated with 0.5 g L^**-1**^ **yeast extract/peptone, 5 g L**^**-1**^ **glucose in the feed and a dilution rate of D = 0.1 h**^**-1**^ *V. criceti*, but not *L. zeae*, was washed out from the bioreactor. Neither acetic nor propionic acid was produced. Obviously complex nutrients are strongly limited. *L. zeae* did grow, but the final lactic acid concentration was very low (0.3 g L^**-1**^), glucose was not consumed completely. This shows the high nutrient demand from both bacteria with an advantage to survive for *L. zeae* under these cultivation conditions.

After this initial experiment the yeast extract/peptone (YP)-concentration was increased to 3 g L^**-1**^, the lactic acid concentration increased due to production by *L. zeae* to 3 g 3 g L^**-1**^, but still glucose was not completely consumed. To ensure anaerobic conditions by reducing the redox potential (reactor was not sparged with nitrogen), cystein was added, but this did not influence biomass and lactate production.

This changed clearly after a second inoculation of *V. criceti* and led to steady state (ST) 1 (only coculture steady states are considered). Now the concentration of complex nutrients was high enough for the cultivation of both organisms simultaneously. Glucose and consequently produced lactic acid were converted to propionic acid. The propionate/acetate-ratio was 1.29 mol mol^**-1**^.

**Figure 2:**
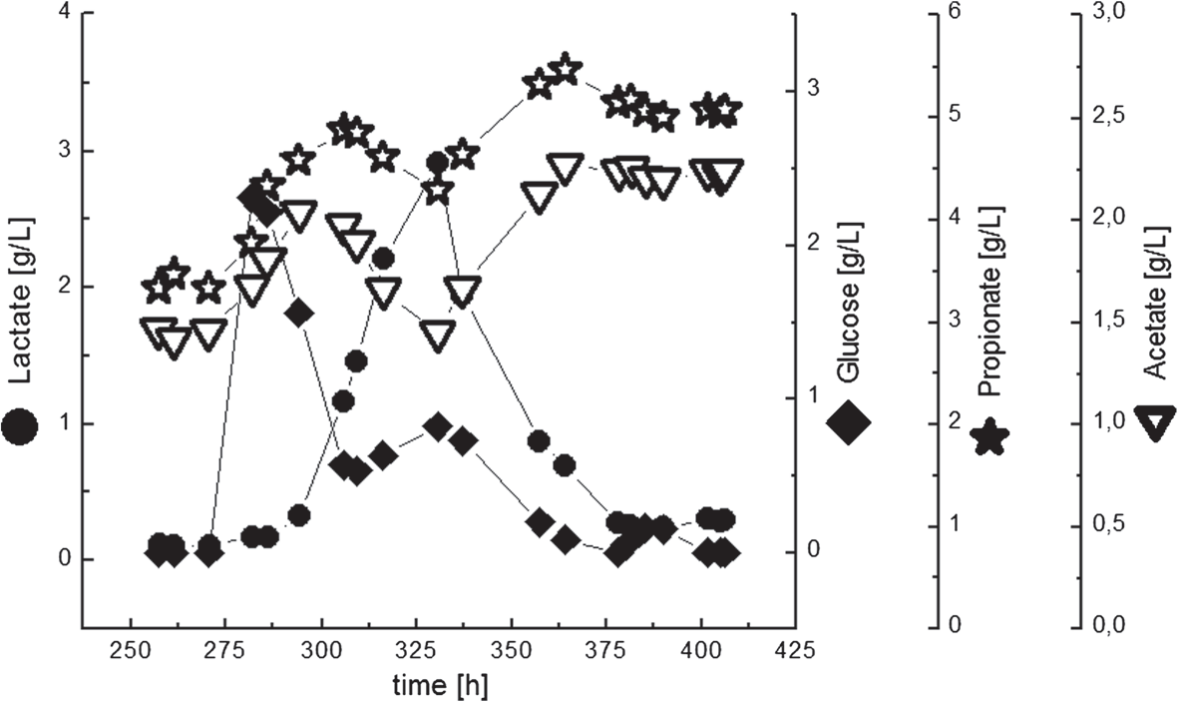
**Section of the CSTR experiment with the co-culture of *L. zeae * and *V. criceti *.** At constant dilution rate and YP-concentration the glucose concentration in the feed was doubled (shift from ST 1 to ST 2, see Table 2). *L. zeae* adapted faster to the new growth conditions (phase 1), but after more than 10 medium exchanges a stable steady state was reached.

After the glucose concentration in the feed was doubled, the mixed culture was strongly influenced (Figure 2, ST 2). Since lactic acid concentration increased, *L. zeae* adapted faster to the new growth conditions (phase 1, Figure 2). *V. criceti*, most probably due to its lower maximal growth rate, converted this lactic acid with a time delay (phase 2, Figure 2).

At the half residence time (5 h, ST 3) glucose consumption was incomplete, but the produced lactic acid was completely converted in acetic and propionic acid. The reason for the incomplete glucose consumption was a nutrient limitation, as can be seen after doubling the YP-concentration in the feed (ST 4). The glucose concentration was reduced from 6 to 2 g L^**-1**^ by consumption of *L. zeae*. Lactic acid again was completely consumed.

The glucose was entirely converted to lactic acid at a YP-concentration of 9 g L^**-1**^ **(ST 5). The propionate concentration rose to 8 g L**^**-1**^**, the propionate/acetate-ratio was 1.21 mol mol**^**-1**^.

**Figure 3:**
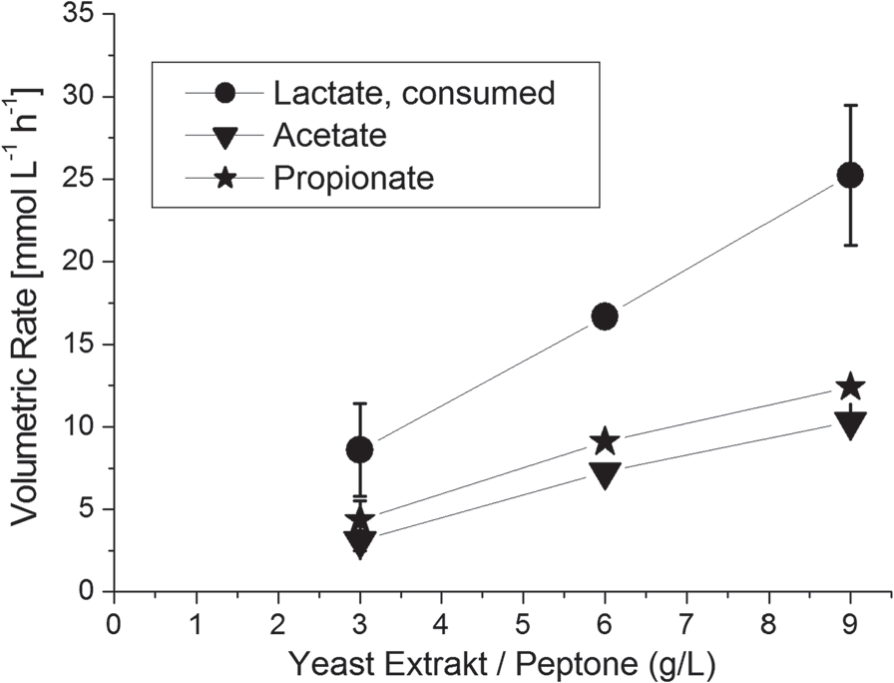
**Volumetric Rates in the defined mixed culture of *L. zeae * and *V. criceti *.**

The last steady state (doubling of the glucose in the feed, ST 6) resulted again in a sequential adaption of *L. zeae* and *V. criceti* as shown in Figure 2. The almost linear correlation between lactate consumption and propionate/acetate production with the concentration of complex nutrients (Figure 3) stresses the high nutrient demand and the double limitation by carbon source and nutrients, respectively.

In an attempt to reach a high propionate concentration, the co-culture fermentation was carried out at a dilution rate of 0.06 h^**-1**^**, and with 5 g L**^**-1**^ **YP and 50 g 50 g L**^**-1**^ glucose in the feed (data not shown). No steady state was reached and strong oscillations were observed. In fact for all the data points given in Table 2 a 10-fold medium exchange was necessary to reach a steady state. Theoretically without any oscillation only four to five medium exchanges should be necessary.

**Table 2:** **Results of CSTR experiments with a defined co-culture of *L. zeae * und *V. criceti * under lactate limitation**

**Steady state**	**YP**	**D**	**Glucose In**	**Glucose Out**	***Δ*Glucose**	**Lactate**	**Acetate**	**Propionate**	**Prop/Ac**
**ST**	**g L**^***-1***^	**h**^***-1***^	**mmol L**^***-1***^	**mmol L**^***-1***^	**mmol L**^***-1***^	**mmol L**^***-1***^	**mmol L**^***-1***^	**mmol L**^***-1***^	**mol mol**^***-1***^
1	3	0.1	27.78	0	27.78	0	24.33	31.49	1.29
2	3	0.1	55.56	0	55.56	0	36.67	54.05	1.47
3	3	0.2	55.56	32.67	22.89	0	16.00	22.70	1.42
4	6	0.2	55.56	13.89	41.67	0	36.33	45.54	1.25
5	9	0.2	55.56	0	55.56	0	51.17	62.16	1.21
6	9	0.2	111.11	40.56	70.56	0	52.00	62.16	1.20

At a YP-concentration of 0.5 g L^**-1**^*V. criceti* was washed out.

### Experiments in dialysis chamber reactor

### *L. zeae* monoculture

**Figure 4:**
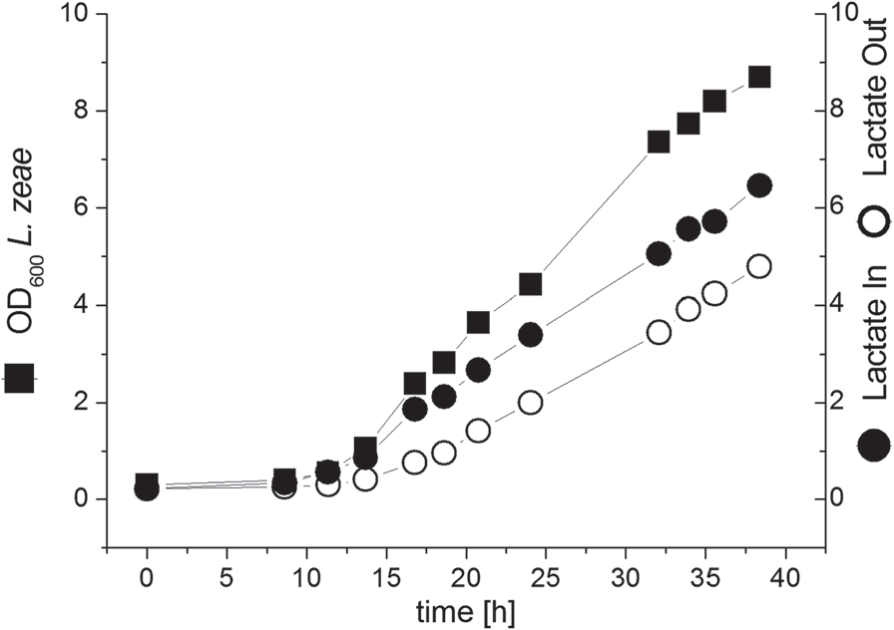
**Cultivation of *L. zeae * in the dialysis reactor.** YP concentration was 3g L^**-1**^.

Cultivation of *L. zeae* in the dialysis reactor showed slow growth and production of lactate (Figure 4). After 37 h of cultivation only 6 g L^**-1**^lactic acid has been produced, the OD was at this time 9. Both values are low in comparison to the mixed culture fermentations in dialysis processing.

### *V. criceti* monoculture

**Figure 5:**
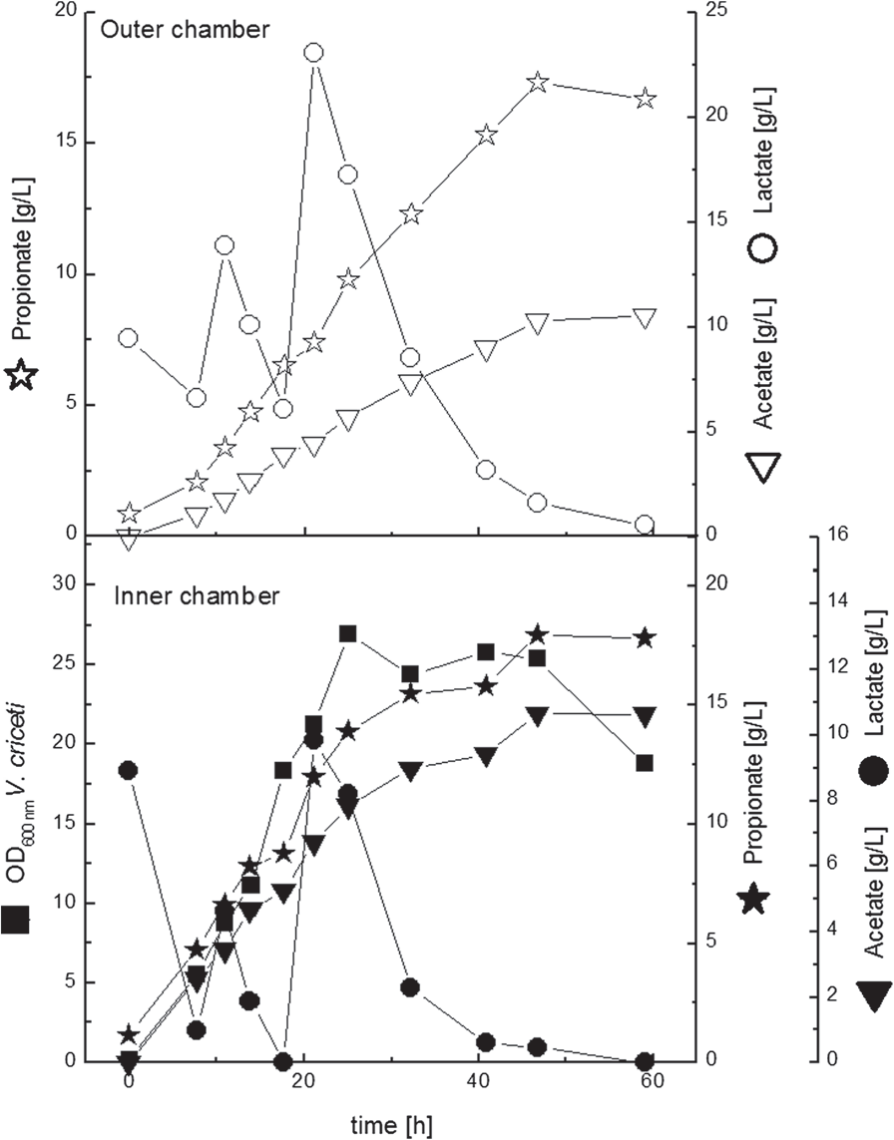
**Cultivation of *V. criceti * in the dialysis reactor.** YP concentration was 3 g L^**-1**^. Lactate was added in pulses due to substrate inhibition of *V. criceti*.

In experiments in serum bottles it turned out, that *V. criceti* is substrate inhibited ( Sabra et al. (2012)). Therefore the initial lactate concentration in the reactor was 20 g L^**-1**^. When lactate was consumed in the inner chamber, it was added in pulses (Figure 5). With this process control a propionate concentration of ∼ 17 g L^**-1**^ **could be achieved, the acetate concentration was ∼ 10 g L**^**-1**^**. This corresponds to a propionate/acetate-ratio of 1.27 mol mol**^**-1**^. The optical density of *V. criceti* was 25, the production rate was high as well (0.39 g L^**-1**^** h**^**-1**^). The production rate of *P. acidiipropionici* usually is much lower (e.g. Sabra et al. (2012)). A summary of the mono- and co-culture experiments in the dialysis reactor is given in Table 3.

**Table 3:** **Results of cultivations of *L. zeae * and *V. criceti * in the dialysis reaktor**

**Dialysis chamber**		**Propionate**		**Prop/Ac**	**Prop/Glu**	**OD**_***Lac***_	**NaOH**	**OD**_***Vei***_
**In**	**Out**	**g L**^***-1***^	**g L**^***-1***^**h**^***-1***^^***a***^	**mol mol**^***-1***^	**mol mol**^***-1***^		**g h**^***-1***^	
3g L^**-1**^ *Yeast extract/Peptone*								
*L. zeae*	\	6^*b*^	0.20^*b*^	\	\	9	2.6	\
*V. criceti*	\	17	0.39	1.27	\	\	0	25
*L. zeae*	*V. criceti*	9	0.39	1.13	1.01	22	13.3	5
*V. criceti*	*L. zeae*	5	0.13	1.4	0.90	5	8	8
10 g L^**-1**^ *Yeast extract/Peptone*								
*L. zeae*	*V. criceti*	19	0.26	1.46	1.17	39	6.8	n.d.^*c*^
*V. criceti*	*L. zeae*	28	0.61	1.47	1.04	19	19.9	39

^**a**^ related to the whole reactor.

^**b**^ value related to lactate.

^**c**^ not detected, cell aggregation of *V. criceti*.

### Experiments with the defined co-culture

**Figure 6:**
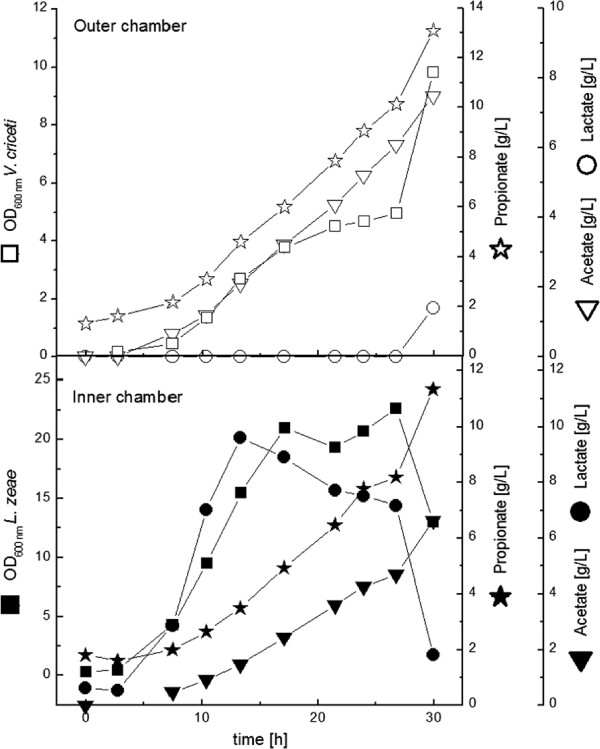
**Cultivation of *L. zeae * (inner chamber) and *V. criceti * (outer chamber) in the dialysis reactor.** YP concentration was 3 g L^**-1**^**. After 30 h there was a membrane rupture, leading to the abrupt change for the last time point. The fermentation was terminated then.**

First *L. zeae* was inoculated in the inner or outer chamber, after base consumption was detected *V. criceti* was inoculated in the other (outer or inner) chamber. This inoculation strategy was followed in all co-culture experiments. YP concentration in the first experiments was 3 g L^**-1**^. Fermentations were stopped, when propionate production in the chamber of *V. criceti* stopped. In the first combination *L. zeae* was inoculated in the inner chamber (Figure 6). With a high growth rate (0.33 h^**-1**^) it reached an OD_600_ of 22, meaning a clear improvement in comparison to the mono-culture experiment. *V. criceti* in the outer chamber grew comparably to the corresponding mono-culture experiment (0.22 h^**-1**^**, OD**_**600**_ **of 5 after 25 h). A membrane rupture after 30 h led to the stop of the fermentation. All glucose was consumed then, lactic acid from the inner chamber could have been converted to propionic/acetic acid in the outer chamber, where lactic acid was limited all the time. Till fermentation termination 9 g L**^**-1**^ **propionic acid with a production rate of 0.39 g L**^**-1**^** h**^**-1**^ **had been produced. This was comparable to the mono-culture experiment. Interesting is the lower propionate/acetate-ratio of 1.13 mol mol**^**-1**^.

**Figure 7:**
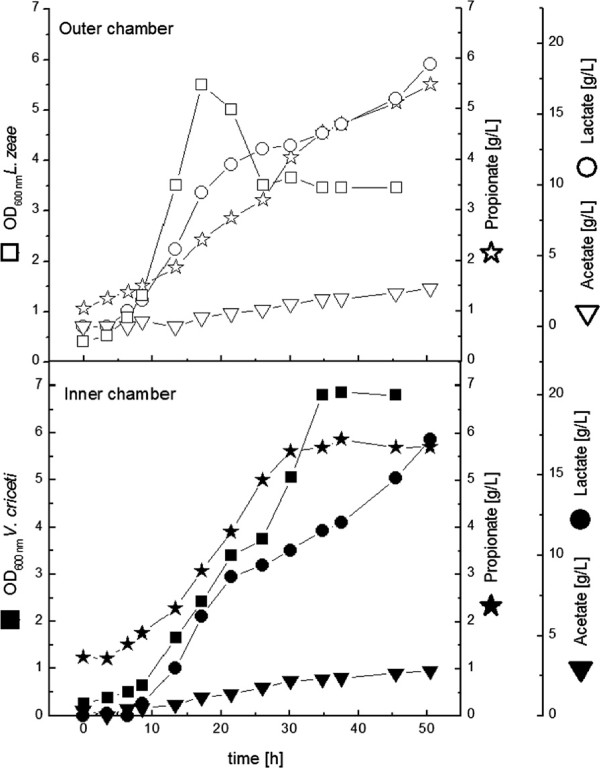
**Cultivation of *V. criceti * (inner chamber) and *L. zeae *** **(outer chamber) in the dialysis reactor.** YP concentration was 3g L^**-1**^.

With *L. zeae* in the outer chamber the OD_**600**_ of *V. criceti* reached a value of 8 (Figure 7). Since the volume of the outer chamber is four times more than in the inner chamber, the total biomass of *V. criceti* was reduced in comparison to the previous experiment. The propionate production rate (0.13 g L^**-1**^ **h**^**-1**^) and the final propionate concentration (5 g L^**-1**^) were lower as well. In comparison to the mono-culture experiment a more effective propionate production (propionate/acetate ratio 1.40 mol mol^**-1**^) was attended with a worse biomass production. In this fermentation the lactic acid concentration in the *V. criceti*-chamber was never limited. When the growth of *V. criceti* stopped, the concentration was 10 g L^**-1**^, a non-inhibiting concentration.

Even after growth termination ($\frac{\text{dX}}{\text{dt}}=0$) of *L. zeae*, lactic acid production proceeded non-growth associated. Lactic acid production in *L. zeae* followed the well-known Luedeking-Piret-kinetics ( Luedeking and Piret (1959)):

$$\frac{\text{dP}}{\text{dt}}=\alpha \cdot \frac{\text{dX}}{\text{dt}}+\beta \cdot X$$

P: product; X: biomass. *α*: parameter for growth-associated production. *β*: parameter for non-growth associated production.

### Improvement of dialysis chamber experiments

In the experiments above neither glucose nor lactic acid have been completely consumed. To improve the co-culture experiments in this respect it was therefore decided to increase the concentration of complex nutrients was increased to 10 g L^**-1**^.

**Figure 8:**
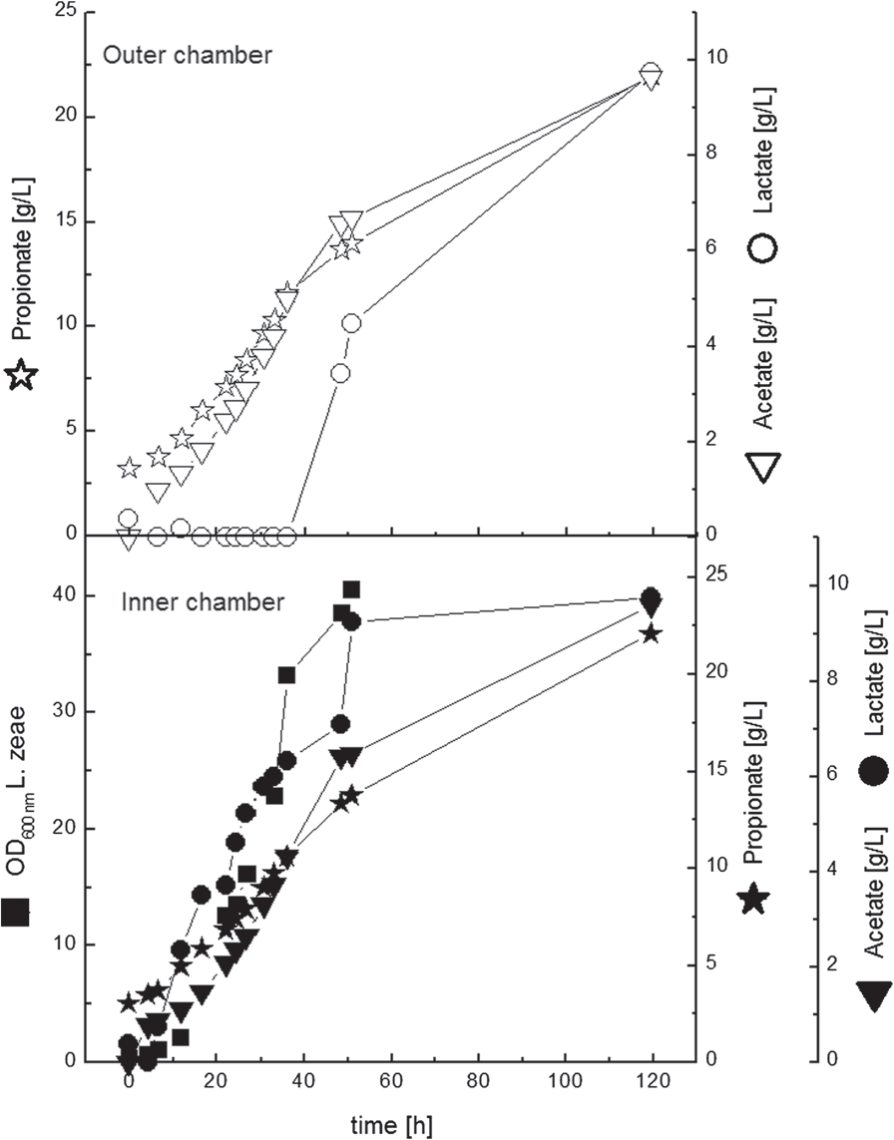
**Cultivation of *L. zeae * (inner chamber) and *V. criceti * (outer chamber) in the dialysis reactor.** YP concentration was 10 g L^**-1**^.

With *V. criceti* in the outer chamber the final propionate concentration was elevated significantly to 19 g L^**-1**^ (Figure 8). The maximal production rate was 0.26 g 0.26 g L^**-1**^ **h**^**-1**^ and the molar ratio of propionate to acetate was 1.46. Whereas the productivity in comparison to the corresponding experiment with lower YP-concentration decreased, the final propionate concentration increased as well as the propionate/acetate-ratio.

Again lactic acid was not completely consumed. This was surprising since a nutrient limitation was implausible due to the high YP-concentration.

**Figure 9:**
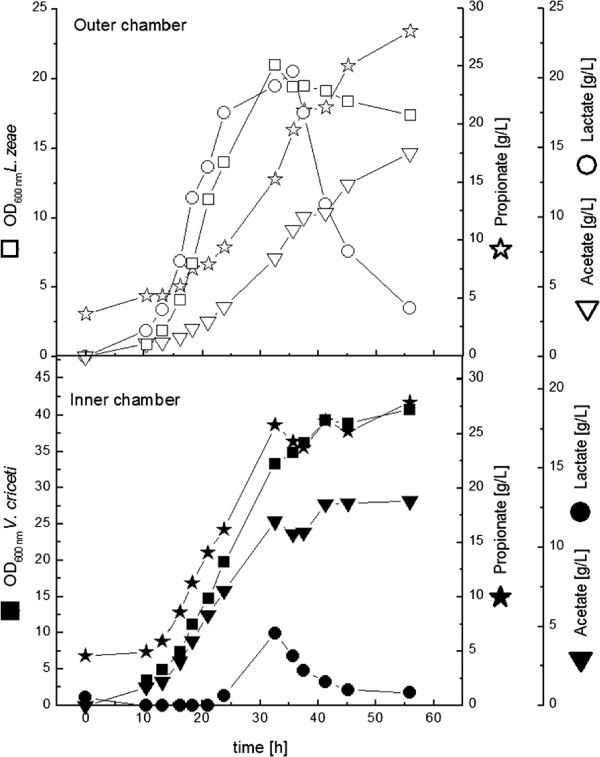
**Cultivation of *V. criceti * (inner chamber) and *L. zeae * (outer chamber) in the dialysis reactor.** YP concentration was 10 g L^**-1**^.

Finally the co-culture combination with *L. zeae* in the outer chamber and *V. criceti* in the inner chamber with high nutrient supply was examined (Figure 9).

Preeminent result is the very high propionate production rate of 0.61 g L^**-1**^ **h**^**-1**^**. Between the 13th and 33rd hour 20 g L**^**-1**^ propionic acid has been produced in the inner chamber.

In this experiment not only the propionate production rate, but the final biomass and propionate/acetate ratio was higher than in the monoculture experiment.

In addition to the results given in Table 3 the propionate-glucose-yield (mol mol^**-1**^) is shown. Theoretically it should be 1.33 mol mol^**-1**^.

$$\begin{array}{llll} C_{6}H_{12}O_{6} & \;\to \;2\;C_{3}H_{6}O_{3}\; & \; & \;\\ \text{in}\;\text{L. zeae} & \; & \; & \;\\ 2\;C_{3}H_{6}O_{3} & \;\to \;\frac{4}{3}\;C_{3}H_{6}O_{2}+\frac{2}{3}\;C_{2}H_{4}O_{2}+\frac{2}{3}\;{\text{CO}}_{2}\; & \; & \;\\ \text{in}\;\text{V. criceti} & \; & \; & \; \end{array}$$

Since the propionate/acetate-ratio was in no experiment 2 mol mol^**-1**^, the maximal propionate/glucose-yield could not be reached. Interestingly both ratios were not directly correlated. Obviously growth and substrate consumption in *L. zeae* depends on the complex nutrient availability which is influenced by the co-culture partner.

## Discussion

In this study a defined co-culture of a lactic acid bacterium, *L. zeae*, and a propionate producer, *V. criceti*, has been realized and investigated for the first time in a dialysis chamber reactor.

Following the classic Methyl-Malonyl-CoA pathway, the ratio should be 2.0 mol mol^**-1**^ (see equation below).

$$\begin{array}{lll} & 3\;{\text{CH}}_{3}\text{CHOHCOOH}\to \; & \;\\ & 2\;{\text{CH}}_{3}{\text{CH}}_{2}\text{COOH}+{\text{CH}}_{3}\text{COOH}+{\text{CO}}_{2}\; & \; \end{array}$$

In CSTR experiments an almost linear correlation of production rate and complex nutrient supply was shown (Figure 3). This may be used for further improvement of the process. Perhaps the use of very high concentrations of complex nutrients may lead to a high propionate concentration in CSTR experiments. From the industrial point of view then the economic aspect would become less attractive. For high titers batch or fed-batch processes, perhaps with cell immobilization seem much more favourable to this end ( Colomban et al. (1993)).

In the dialysis cultures with high nutrient supply very high propionate production rates (0.61 g L^**-1**^ **h**^**-1**^) with final titers up to 28 g L^**-1**^ have been realized.

**Figure 10:**
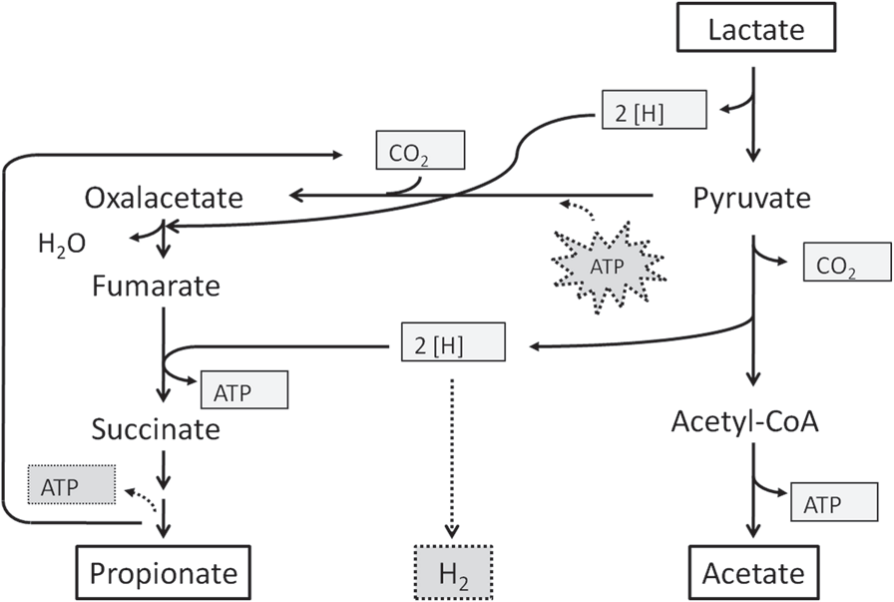
**Simplified scheme of propionate metabolism in *Veillonella * spec.: In contrast to *Propionibacterium * spec., in *Veillonella * spec. there is no *transcarboxylase *.** The transcarboxylation is realized via a *pyruvate-carboxylase* and a *methyl-malonyl-CoA-decarboxylase*. This is less energy efficient. 1 mol ATP is consumed by the pyruvate-carboxylation, two third can be recovered by the fumarat-reduction, one third via methylmalonyl-CoA-decarboxylation. As in the acryloyl-pathway there is no net ATP production. The regeneration of NADH_2_ to NAD can be realized by hydrogen production, which is more energy efficient ( Denger and Schink (1992)).

An interesting result was the propionate/acetate ratio, that never was 2 mol mol^**-1**^ and varied strongly in dependance of the co-culture experiment. In general the Methyl-Malonyl-CoA-pathway is available for lactic acid conversion in *Veillonella* spec.. *V. criceti* has no *transcarboxylase*, but a *pyruvate-carboxylase* and a *methyl-malonyl-CoA-decarboxylase* to transfer the carbon dioxide to pyruvate (Figure 10). The carboxylation consumes energy, which is not completely recovered by the decarboxylase via sodium ion-translocation Seeliger et al.(2002). So the following equation and energy balance can be generally applied to *Propionibacterium* spec., but not to *V. criceti*:

$$\begin{array}{lll} & 3\;{\text{CH}}_{3}\text{CHOHCOOH}\to \; & \;\\ & 2\;{\text{CH}}_{3}{\text{CH}}_{2}\text{COOH}+{\text{CH}}_{3}\text{COOH}+{\text{CO}}_{2}\; & \;\\ & \Delta {G^{0}}^{\prime }=-56,67\;\text{kJ/mol lactate}\; & \; \end{array}$$

In *Veillonella parvula* this pathway is less effectiv than in *Propionibacterium freudenreichii*. *V. parvula* gets 0.33 mol ATP per mol lactate, *P. freudenreichii* 0.78 mol ATP ( Seeliger et al. (2002)).

This less effective energy recovery compensates *Veillonella* spec. by higher growth and substrate consumption rates ( Seeliger et al. (2002)).

Another possibility for the compensation in *Veillonella* spec. is the production of hydrogen, resulting in a reduction of the energy ineffective carboxylation. Denger and Schink (1992) proposed the following reaction equation:

$$\begin{array}{lll} & 10\;{\text{CH}}_{3}\text{CHOHCOOH}\to 6\;{\text{CH}}_{3}{\text{CH}}_{2}\text{COOH}+\; & \;\\ & 4\;{\text{CH}}_{3}\text{COOH}+4\;{\text{CO}}_{2}+2\;H_{2}+2\;H_{2}O\; & \;\\ & \Delta {G^{0}}^{\prime }=-51,8\;\text{kJ/mol lactate}\; & \; \end{array}$$

The energy yield per mol lactate is then comparable in both organisms. When the nutrients are limited, the hydrogen production rises as can be seen from both the CSTR and the dialysis reactor experiments. Although we did not measure hydrogen directly we could not detect any other metabolite by HPLC. Hydrogen production explains well our results in agreement with similar investigations ( Denger and Schink (1992); Seeliger et al. (2002)).

Both organisms have a high nutrient demand. In the experiment with a low nutrient supply, the faster growing *L. zeae* outcompetes *V. criceti* for the complex nutrients. *V. criceti* has a high demand for complex nutrients as vitamins, amino acids and even nucleobases ( Durant et al. (1997)). For some of those nutrients *V. criceti* has obviously a higher demand than *L. zeae*, since *L. zeae* can grow well on hydrolyzed wheat straw, but *V. criceti* cannot ( Sabra et al. (2012)).

The metabolism of *L. zeae* is markedly stimulated by the co-culture partner independent of nutrient limitations and experimental setup. This behaviour could be investigated in more detail in the dialysis chamber reactor due to the local separation of both microorganisms. A reason for the strong improvement of the growth of *L. zeae* may be, that *V. criceti* reduces the redox potential of the medium by hydrogen production. The reduced redox potential may have a strong impact on microorganisms ( van Hoek and Merks (2012)).

Surprisingly *V. criceti* stops growth in the co-culture experiment with high nutrient supply when it is cultured in the outer chamber - although carbon and most probably complex nutrients are available. We suggest the accumulation of an inhibiting secondary metabolite, that develops its inhibiting potential not until a critical concentration is reached, probably comparable to *quorum sensing*-signals. This interesting phenomenon should be investigated in future studies in more details. The dialysis chamber reactor is ideal for the investigation of such phenomena which are based on low molecular weight “communication molecules”. The communication molecule may become only relevant at higher cell densities.

Investigations in the dialysis reactor in CSTR will give more information. Still the experimental requirements, especially for medium (50 L for one steady state assuming 10 medium exchanges as shown in the continuous culture), are very high and we now undergo efforts to realize these experiments.

The overall strength of this system for co-culture investigations is the local separation of microorganisms. This allows not only the investigation of growth behaviour as performed in this study, but also a detailed observation of a singularised mircroorganism by genomic, proteomic and transcriptomic approaches.

With these data, mathematical models can be established to describe the kinetics of cell growth and metabolism of the defined microbial community. For a more fundamental understanding of the microbial community, intracellular metabolic fluxes should be estimated. Metabolic fluxes of microbial communities have been seldom studied, therefore the existing methods for flux estimation need to be adapted and further developed. The results from kinetic and flux analysis should help to identify possible limiting step(s) and key parameters for the development and optimization of the novel bioprocesses for propionic acid production.

## Competing interests

The authors declare that they have no competing interests.
